# A comprehensive characterization of the impact of mycophenolic acid on the metabolism of Jurkat T cells

**DOI:** 10.1038/s41598-017-10338-6

**Published:** 2017-09-05

**Authors:** Ana A. Fernández-Ramos, Catherine Marchetti-Laurent, Virginie Poindessous, Samantha Antonio, Céline Petitgas, Irène Ceballos-Picot, Pierre Laurent-Puig, Sylvie Bortoli, Marie-Anne Loriot, Nicolas Pallet

**Affiliations:** 1 INSERM UMR-S 1147, Centre Universitaire des Saints-Pères, 45 rue des Saints-Pères, 75006 Paris, France; 2 Université Paris Descartes, Sorbonne Paris Cité. 45, rue des Saints-Pères, 75006 Paris, France; 3 INSERM UMR-S 1124, 45 rue des Saints-Pères, 75006 Paris, France; 4 Assistance Publique-Hôpitaux de Paris, Hôpital Européen Georges Pompidou, Service de Biochimie, 20 rue Leblanc, 75015 Paris, France; 5 Assistance Publique-Hôpitaux de Paris, Hôpital Necker-Enfants Malades, Laboratoire de Biochimie métabolomique et protéomique, 149 rue de Sèvres, 75015 Paris, France

## Abstract

Metabolic reprogramming is critical for T cell fate and polarization and is regulated by metabolic checkpoints, including Myc, HIF-1α, AMPK and mTORC1. Our objective was to determine the impact of mycophenolic acid (MPA) in comparison with rapamycin (Rapa), an inhibitor of mTORC1, on the metabolism of Jurkat T cells. We identified a drug-specific transcriptome signature consisting of the key enzymes and transporters involved in glycolysis, glutaminolysis or nucleotide synthesis. MPA produced an early and transient drop in the intracellular ATP content related to the inhibition of *de novo* synthesis of purines, leading to the activation of the energy sensor AMPK. MPA decreases glycolytic flux, consistent with a reduction in glucose uptake, but also in the oxidation of glutamine. Additionally, both drugs reduce aerobic glycolysis. The expression of HIF-1α and Myc, promoting the activation of glycolysis and glutaminolysis, was inhibited by MPA and Rapa. In conclusion, we report that MPA profoundly impacts the cellular metabolism of Jurkat T cells by generating an energetic distress, decreasing the glycolytic and glutaminolytic fluxes and by targeting HIF-1α and Myc. These findings open interesting perspectives for novel combinatorial therapeutic strategies targeting metabolic checkpoints to block the proliferation of T cells.

## Introduction

Over the recent years, the understanding of the relationships between metabolism and immune cell activation (i.e., the immunometabolism), proliferation and polarization has significantly progressed. It is now widely accepted that T cell metabolism, a highly dynamic and plastic process responding to environmental cues to fine tune cellular functions, plays a key role in the orchestration of adaptive immune reactions. Upon antigen stimulation, metabolic reprogramming is engaged through aerobic glycolysis (the transformation of glucose into pyruvate and lactate, also known as the Warburg effect), glutaminolysis (the transformation of glutamine into the anaplerotic compound α-ketoglutarate), and the production of biosynthetic precursors such as pyrimidine and purine nucleotides, lipids and amino acids by the pentose phosphate and serine pathways to sustain rapid growth and proliferation of T cells^1–4^. Whereas naïve, regulatory and memory T cells rely on oxidative phosphorylation (OXPHOS) to produce ATP, the switch for aerobic glycolysis^5–10^, a process much less efficient for ATP production than OXPHOS^1, 2, 6^, is critical not only for T cell activation and proliferation but also for CD8^+^ cytotoxic and T helper 17 (Th_17_) cells maintenance and function^7, 11^. Metabolic reprogramming is under the control of molecular sensors that integrate both activating extracellular signals from the microenvironment and the intracellular energetic status. These “metabolic checkpoints” comprise Akt (protein kinase B, PKB), mechanistic Target of Rapamycin Complex 1 (mTORC1), AMP-activated protein kinase (AMPK), Myc and Hypoxia Inducible Factor (HIF)-1α^1, 2^, which are not only the mediators of upstream messages that foster metabolic reprogramming but also critical for licensing T cell polarization into specific lineages, e.g., HIF-1α for Th_17_ cells or mTOR for T_reg_ differentiation^9^.

Indirect evidence supports the notion that immunosuppressive molecules targeting key signalling pathways for the activation and proliferation of T cells can also modulate the activity of metabolic checkpoints and impact metabolic reprogramming^12–14^. Thus, the inhibition of mTORC1 by rapamycin (Rapa) promotes both immunosuppression and profound metabolic changes^15, 16^. In addition to the fundamental role of mTORC1 in controlling the metabolic reprogramming of immune cells, emerging data indicate that drugs that modulate purine synthesis also affect the metabolic checkpoints and impact cell metabolism. In particular, mycophenolic acid (MPA) is likely to have an impact on T cell metabolism. MPA reversibly inhibits inosine monophosphate dehydrogenase (IMPDH) type II, a rate-limiting enzyme involved in *de novo* purine synthesis, to decrease the guanosine pool and DNA synthesis. Consequently, MPA selectively blocks the proliferation of lymphocytes because they rely more on *de novo* pathway than on the salvage pathway^17–22^. In agreement with its role in metabolic checkpoints and metabolic reprogramming, MPA modifies the activities of the Myc and HIF-1α signalling pathways in endothelial cells^23^, affects the proliferation of gastric cancer cells in a PI3K-AKT-mTOR pathway-dependent manner^24^, and promotes T cell anergy and metabolic reprogramming in the CD4^+^ T cells via suppression of the Akt/mTOR and STAT5 pathways^25, 26^. Therefore, elucidating the consequences of the metabolic changes that are induced by immunosuppressive drugs on immunity beyond their well-described effects is an attractive research avenue. The results from such studies could lead to the discovery of novel paradigms for the use of immunosuppressive drugs.

In the present study, we performed a comprehensive characterization of the metabolic activities in proliferating human T cells (Jurkat T cells) exposed to MPA or rapamycin. Our results indicate that MPA profoundly impacts the energetic status of the cell and alters the metabolism of glucose and glutamine through the down-regulation of the metabolic checkpoints HIF-1α and Myc. One could therefore speculate that the immunosuppressive activity and/or the side effects of MPA are, at least in part, related to the drug-induced modifications in the metabolism of T cells.

## Results

### MPA decreases cell viability and promotes apoptosis

**Figure 1:**
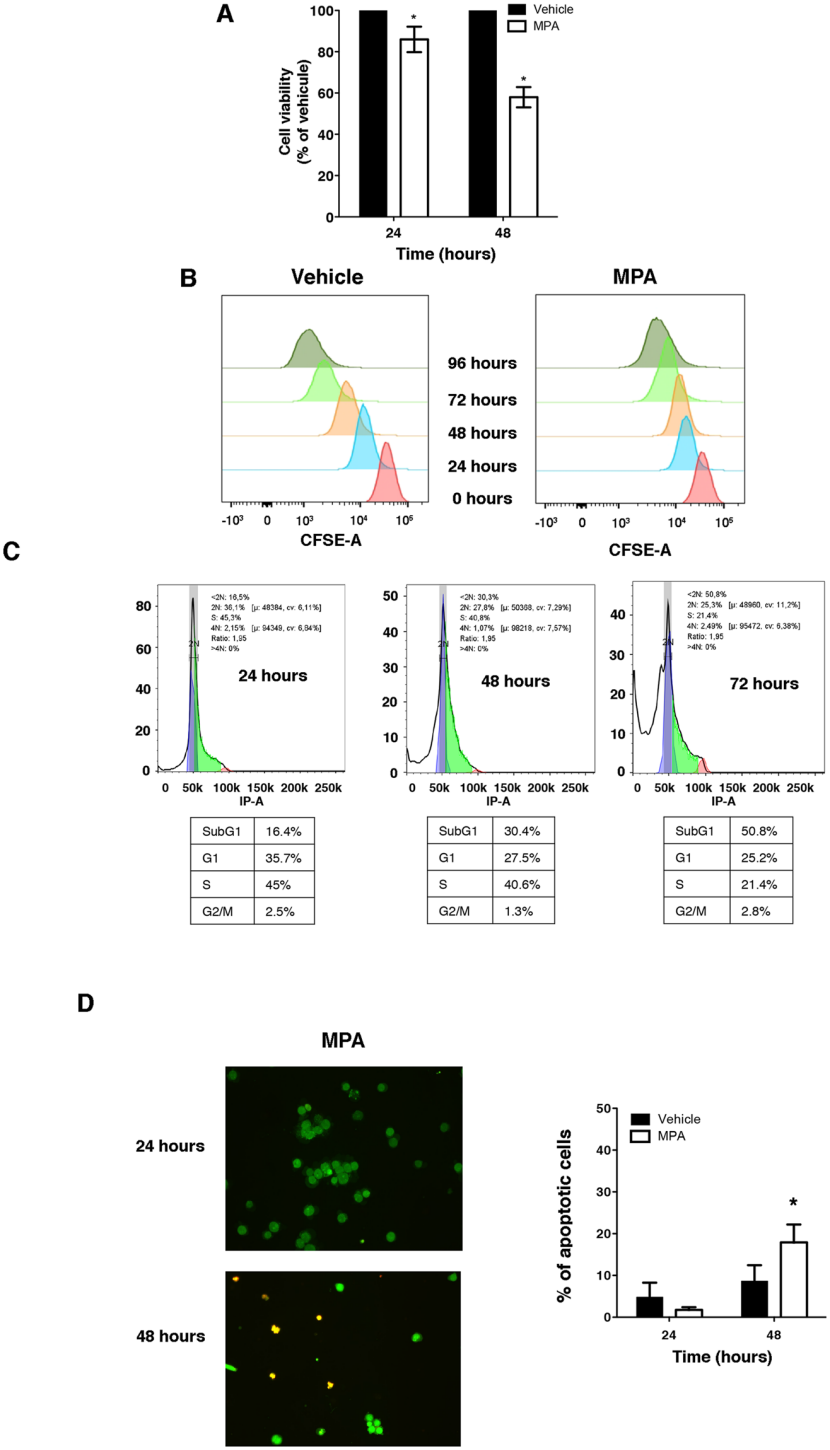
Mycophenolic acid blocks T cell proliferation and promotes apoptosis. (**A**) Cell viability after incubation with 0.5 µM MPA or vehicle for 24 h and 48 h. The data are from four independent experiments. Mann-Whitney U test: *P < 0.05. (**B**) Cell proliferation of CFSE-stained Jurkat cells. The cells were incubated with 0.5 µM MPA or vehicle for up to 96 h. (**C**) Cell cycle analysis of PI-stained Jurkat cells. The cells were incubated for up to 72 h with 0.5 MPA or vehicle. The data are from three independent experiments. (**D**) (Left) Images representing live cells (green) and apoptotic cells (red) after 24 h and 48 h of incubation with 0.5 µM MPA. (Right) Histograms representing the percentage of apoptotic cells after 24 h and 48 h exposure to vehicle or MPA. The data are from three independent experiments.

To define the impact of MPA on the metabolism of proliferating T cells, we incubated Jurkat T cells (a validated model for studying the metabolism of proliferating T cells^27–30^) with a low concentration (0.5 µM) of MPA (i.e., 0.16 μg/ml) for up to 48 h (the doubling time of Jurkat cells being ≈24 h). At this concentration, MPA significantly decreased cell viability to approximately 40% after 48 h (Fig. 1A). Since a reduction in cell viability can result from reduced proliferation and/or increased cell death, we assessed the impact of MPA on cell cycle and apoptosis. MPA reduced cell proliferation in a time-dependent manner based on the measurement using carboxyfluorescein succinimidyl ester (CFSE) (Fig. 1B). In line with the reduction in proliferation, MPA promoted an accumulation of cells stalled in sub-G1 phase in a time-dependent manner, with 50% of cells in the sub-G1 phase after 72 h compared with 17% sub-G1 cells in the vehicle-treated cells (data not shown). This suggests that MPA induces alterations in the cell cycle by arresting the cells at sub-G1 phase (Fig. 1C). In addition to promoting cell cycle arrest, we observed a time-dependent increase in apoptosis, and after incubation for 48 h with MPA, 15% of the cells were apoptotic (Fig. 1D). This confirms that MPA, even at low dose, promotes apoptosis^12, 31–33^. Even if a small proportion of cells are killed by MPA, this proportion is low and the biological effects that we describe reflect the metabolism of living cells and are not blurred by dying cells.

### MPA impacts the expression of genes involved in glycolysis, glutaminolysis and nucleotide synthesis

**Figure 2:**
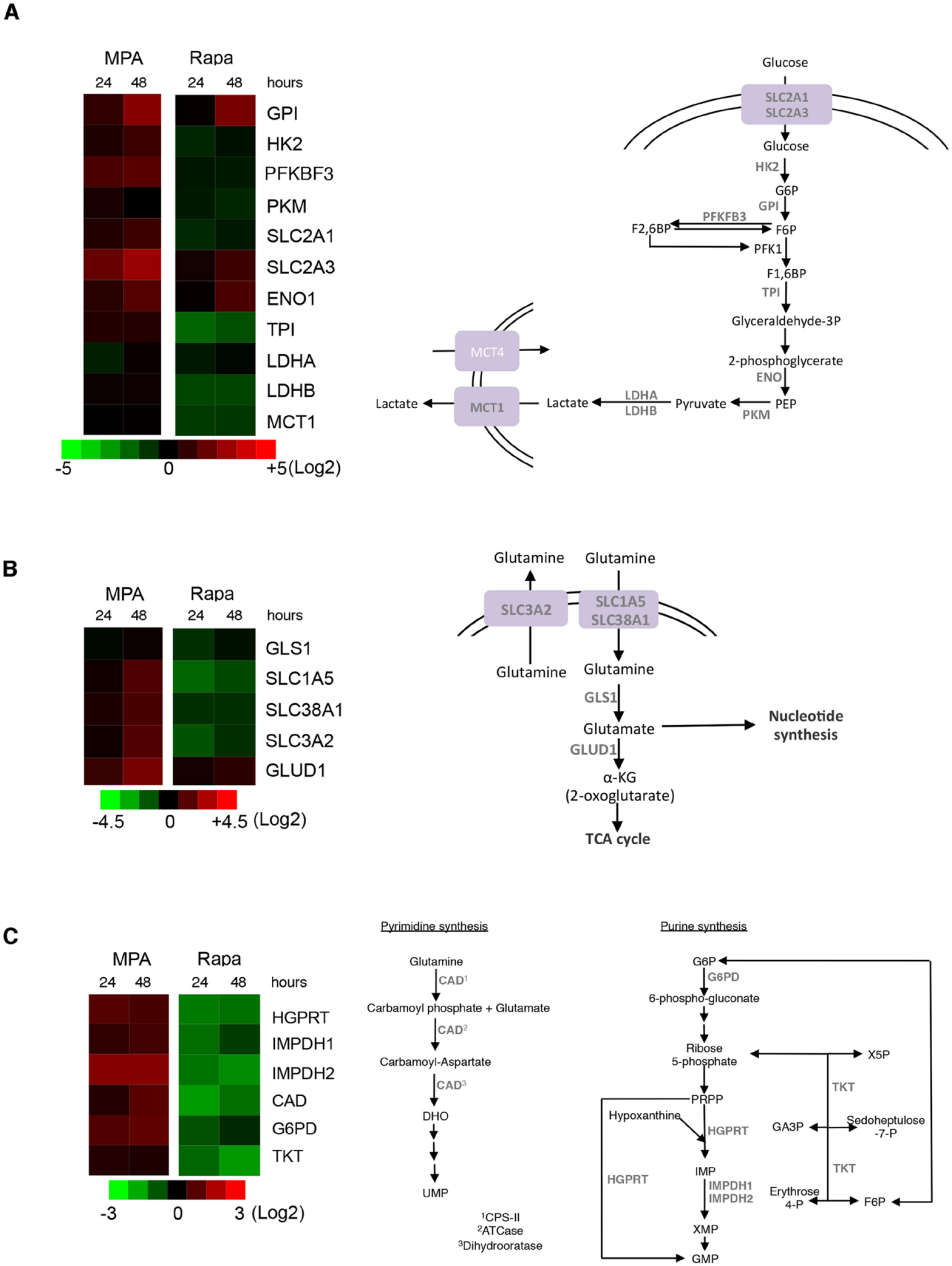
MPA modifies the expression of genes implicated in glycolysis, glutaminolysis and nucleotide synthesis. Left, Heat map representation of the expression of genes involved in glycolysis (**A**), glutaminolysis (**B**) and nucleotide synthesis (**C**) after treatment with 0.5 µM MPA or 5 µM rapamycin for 24 h and 48 h and analysis by qRT-PCR. Right, the schematic representation of the analysed genes and their respective biochemical pathways. The data are from three independent experiments. ATCase: aspartate carbamoyltransferase; CAD: carbamoyl-phosphate synthetase 2, aspartate transcarbamylase, and dihydroorotase; CPS-II: carbamoyl phosphate synthetase II; DHO: dihydroorotate; ENO1: enolase 1; F1,6BP: fructose1,6-biphosphate; F6P: fructose-6-phosphate; G6P: glucose-6-phosphate; G6PD: glucose-6-phosphate dehydrogenase: GA3P: glyceraldehyde 3-phosphate; GLS1: glutaminase 1; GLUD1: glutamate dehydrogenase 1; GMP: guanosine monophosphate; GPI: glucose-6-phosphate isomerase; HGPRT: hypoxanthine-guanine phosphoribosyltransferase; HK2: hexokinase II; IMP: inosine monophosphate; IMPDH1: inosine 5′-monophosphate dehydrogenase 1; IMPDH2: inosine 5′-monophosphate dehydrogenase 2; LDHA: lactate dehydrogenase A; LDHB: lactate dehydrogenase B; MCT1: monocarboxylate transporter 1; MCT4: monocarboxylate transporter 4; PEP: phosphoenolpyruvate; PKFKB3: 6-phosphofructo-2-kinase/fructose-2,6-biphosphatase 3; PKM: pyruvate kinase muscle; PRPP: phosphoribosyl pyrophosphate; SLC1A5: solute carrier family 1, member 5; SLC2A1: solute carrier family 2, member 1; SLC2A3: solute carrier family 2, member 3; SLC38A1: solute carrier family 38, member 1; SLC3A2: solute carrier family 3, member 2; TCA cycle: tricarboxylic acid; TKT: transketolase; TPI: triosephosphate isomerase; UMP: uridine monophosphate; ×5 P: xylulose-5-phosphate.

To test whether MPA impacts the metabolism of proliferating T cells, we performed a transcriptomic analysis of the expression of genes encoding enzymes and transporters involved in glycolytic and glutaminolytic fluxes (Fig. 2A and B) as well as nucleotide synthesis (Fig. 2C) and playing a major role in these processes. We compared the gene expression signature of the MPA-treated T cells with cells that were incubated with 5 μM rapamycin (Rapa), an inhibitor of the metabolic checkpoint mTORC1, which we used as the control. Strikingly, MPA and Rapa generated highly different expression profiles. Rapa treatment was associated with a near global shutdown of expression of genes implicated in glutaminolysis and nucleotide synthesis and several genes implicated in glycolysis, consistent with the possible consequence of the global reduction in anabolism and energy expenditure imposed by mTOR inhibition. In contrast, most of these genes were upregulated upon MPA exposure. However, a cluster of genes including glucose-6-phosphate isomerase (GPI), solute carrier family 2, member 3 (SLC2A3), enolase 1 (ENO1) and glutamate dehydrogenase 1 (GLUD1) were upregulated both by MPA and Rapa. These findings indicate that MPA impacts biochemical circuitries supporting cellular metabolism in manner different from Rapa, and this is likely related to the specific pharmacodynamics of each drug. On the other hand, the common pathways are likely activated in response to a similar biological effect.

### MPA impacts the activity of metabolic checkpoints

**Figure 3:**
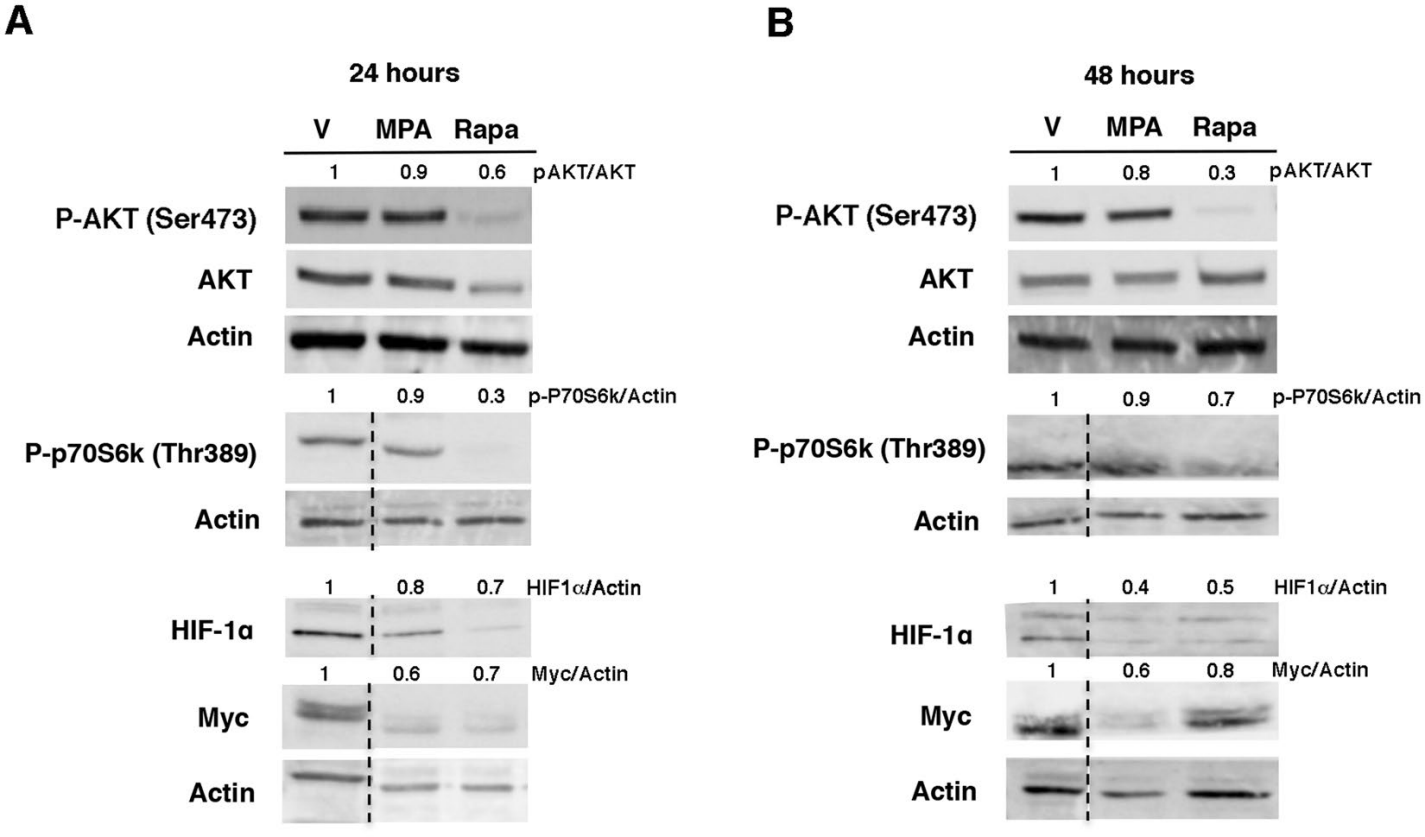
MPA alters the expression of the metabolic checkpoints HIF-1α and Myc. Immunoblot representing phospho-Akt (S473), total Akt, phospho-p70S6K (70 kDa ribosomal protein S6 kinase 1, T389), HIF-1α (hypoxia inducible factor 1α), Myc and actin levels after 24 h (**A**) or 48 h (**B**) of treatment with 0.5 µM MPA or 5 µM rapamycin. The immunoblot is representative of three independent experiments. The dotted line indicates that some non-relevant lanes were cut.

Having provided evidence that MPA and Rapa have different effects on the expression of genes involved in critical metabolic pathways including glutaminolysis and glycolysis, we next evaluated if MPA influences the activity of the metabolic checkpoints that shape the metabolism of lymphocytes such as HIF-1α and Myc and their upstream activators, Akt and mTOR^11, 34^. Indeed, we observed that MPA reduces the expression of the transcription factors HIF-1α and Myc without altering the activities of mTORC1 (monitored with p70S6K phosphorylation at threonine 389) and Akt (which requires phosphorylation at serine 473 to be fully activated), thereby suggesting that MPA decreases the expression of Myc and HIF-1α independently of the Akt and mTOR signalling pathways (Fig. 3 and Supplementary Figure 1). In line with the results of the comparative transcriptomic analysis that revealed differential gene signatures of MPA and Rapa, Rapa was mostly associated with a global reduction of the activities of the metabolic checkpoints tested (Akt, mTORC1, HIF-1α and Myc) consistent with a global inhibition of the expression of genes involved in glycolysis and glutaminolysis (Fig. 2). Notably, despite a reduced expression of HIF-1α and Myc, genes regulating glycolysis and glutaminolysis, and which are, at least in part, under the control HIF-1α and Myc, were expressed, suggesting that redundant signalling pathways under the control of Akt and/or mTOR and independent of HIF-1α and Myc remain activated.

### MPA reduces the intracellular ATP levels and activates AMPK

**Figure 4:**
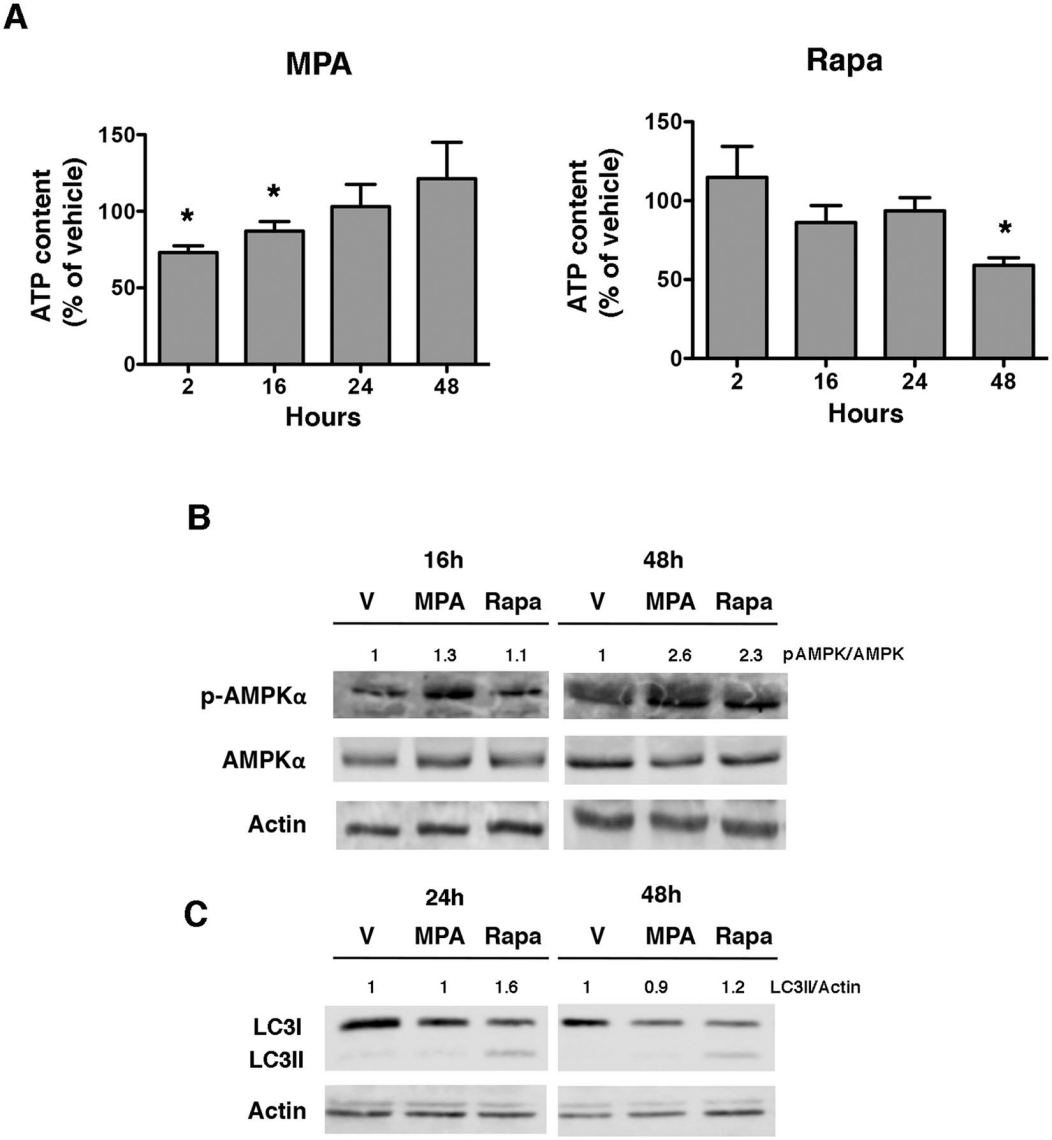
MPA reduces the intracellular ATP content. (**A**) Percentage of ATP in the Jurkat cells incubated with 0.5 µM MPA or 5 µM rapamycin for up to 48 h. The data are from four independent experiments. Mann-Whitney U test: *P < 0.05. (**B**) Immunoblot representing phospho-AMPKα, AMPKα and actin levels after incubation with 0.5 µM MPA, 5 µM rapamycin or vehicle (V). The immunoblot is representative of three independent experiments. (**C**) Immunoblot representing LC3 and actin levels after incubation with 0.5 µM MPA, 5 µM rapamycin or vehicle (V). The immunoblot is representative of three independent experiments.

Since MPA and Rapa have differential impacts on critical metabolic checkpoints and genes regulating glycolysis and glutaminolysis, we hypothesized that these drug-induced effects can lead to the modification of cellular energetic status. We first evaluated the production of ATP in the cells exposed to MPA or Rapa. Indeed, compared with the vehicle-treated cells, Rapa induced a progressive drop in intracellular ATP levels, which became significant after 48 h of drug exposure (with 50% diminution of ATP content) (Fig. 4A). This is consistent with mTORC1 inhibition, as mTORC1 is involved in mitochondrial function and proliferation^35^. Unexpectedly, and in sharp contrast with Rapa, MPA induced an early and moderate drop in the ATP content after 2 h of treatment (with maximal reduction of 25% in the ATP content) for up to 16 h, whereas after 24 h and 48 h of incubation, the ATP levels normalized compared to the levels in the vehicle-treated cells. This result is in agreement with the inhibitory effect of MPA on *de novo* purine synthesis but not on pyrimidine molecules (Supplementary Figure 2), which has a direct consequence on the production of adenosine^36^, and it likely allows for the activation of compensatory mechanisms. To determine if adaptive processes were activated upon MPA-induced ATP starvation, we monitored the activity of AMPK, the master sensor of energetic status of the cell, which is regulated by the ATP/AMP ratio. In concordance with the reduction in the ATP content, the catalytic subunit of AMPK was phosphorylated at threonine 172 after 16 h of incubation with MPA (Fig. 4B) but not with Rapa at the same time point. In line with the presence of an efficient compensatory mechanism for the early energetic distress induced by MPA, autophagy, which is instrumental for adaptation to energetic stress^37, 38^, was not increased by MPA, whereas autophagy was increased after 24 h of incubation with Rapa; this observation indicates that the duration of energetic stress induced by MPA is transient enough to avoid autophagy activation (Fig. 4C). Together, these results suggest that MPA promotes a transient decrease in the levels of ATP followed by the activation of AMPK aimed at restoring the intracellular ATP pool.

### MPA decreases glycolytic and glutaminolytic fluxes

**Figure 5:**
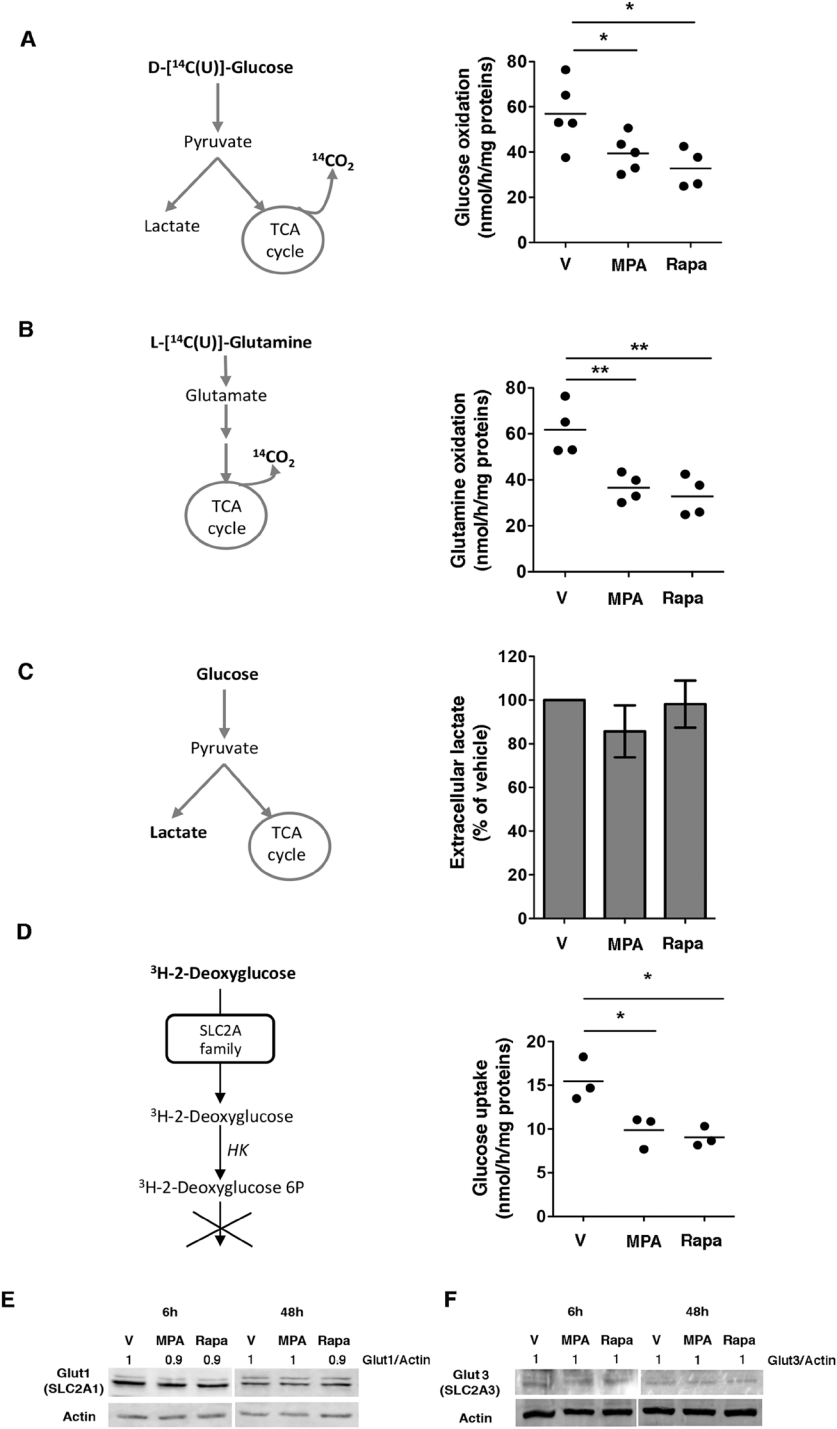
MPA reduces glycolytic and glutaminolytic fluxes. (**A**,**B**) Glycolytic and glutaminolytic fluxes after incubation with 0.5 µM MPA or 5 µM rapamycin for 48 h. Left, schematic illustration of the protocol. Right, dot plots showing glucose oxidation and glutamine oxidation by nmol of glucose or glutamine metabolized per hour per mg of protein. The data are from four to five independent experiments. Mann-Whitney U test: *P < 0.05; **P < 0.001. (**C**) Extracellular lactate production after incubation with 0.5 µM MPA or 5 µM rapamycin for 48 h. Left, schematic illustration of the protocol. Right, histograms showing the percentage of lactate produced by the Jurkat cells. The data are from four independent experiments. Mann-Whitney U test: non-significant. (**D**) Glucose uptake after incubation with 0.5 µM MPA or 5 µM rapamycin for 48 h. Left, schematic representation of the method. Right, glucose uptake analysed by the quantity of^3^H-2-deoxyglucose entering the Jurkat cells. The data are from three independent experiments. Mann-Whitney U test: *P < 0.05. (**E**) Immunoblot of Glut1 (SLC2A1) and actin levels after incubation with vehicle (V), 0.5 µM MPA or 5 µM rapamycin for 6 h or 48 h. The immunoblot is representative of three independent experiments. (**F**) Immunoblot of Glut3 (SLC2A3) and actin levels after incubation with vehicle (V), 0.5 µM MPA or 5 µM rapamycin for 6 h or 48 h. The immunoblot is representative of three independent experiments.

To further characterize the functional consequences of MPA on the metabolism of proliferating T cells, we monitored the effects of MPA on the oxidation of glucose and glutamine. The intensity of these processes is represented by the flux of carbon atoms from glucose or glutamine through glycolysis or glutaminolysis, respectively, towards the TCA (tricarboxylic acid) cycle, which results in the oxidation of these carbon atoms CO_2_. By measuring the rate of release of CO_2_ from U-^14^C-glucose and U-^14^C-glutamine, we observed that glucose oxidation was reduced by nearly 40% after 48 h incubation with MPA, and glutamine oxidation was also reduced to a similar extent (Fig. 5A and B). We observed similar results on glucose and glutamine oxidation fluxes with Rapa. Our results indicate that MPA reduced the ability of Jurkat cells to oxidize glucose and glutamine, associated with the reduction in OXPHOS activity. Since the pyruvate produced by glycolysis can either enter the TCA cycle to result in OXPHOS or be converted to lactate (by lactic acid fermentation), we tested whether the reduction in OXPHOS could be compensated by an increase in the production of lactic acid. However, MPA did not significantly impact the production of extracellular lactate (Fig. 5C). Together, these data indicate that MPA decreases glucose and glutamine oxidation without affecting lactate production.

### MPA decreases glucose uptake

Since MPA reduced glucose utilization in our Jurkat T cell line, we investigated whether this drug can alter glucose influx from the extracellular medium. For that purpose, we monitored the intracellular concentrations of ^3^H-2-deoxyglucose, a radioactive analogue of glucose that is phosphorylated by hexokinases but cannot undergo further glycolysis. MPA significantly decreased the glucose uptake by nearly 50%, suggesting that the reduction of glucose oxidation could result from the reduction of glucose uptake (Fig. 5D). However, the expression of Glut1 (SLC2A1) and Glut3 (SLC2A3), glucose transporters with a selective cell-intrinsic function in the metabolic reprogramming of T cells^39^, was not modified after 6 h (short) or 48 h (long) exposure to MPA (Fig. 5E and F). The mechanism by which MPA reduces glucose uptake without affecting Glut1 and Glut3 transporter requires further investigation. However, it is conceivable that MPA has an indirect functional impact on Glut, which mediates ATP-dependent secondary active transport.

## Discussion

In this study, we provide a comprehensive description of the profound metabolic changes in response to MPA treatment in proliferating immortalized T cells. MPA promotes a general rewiring of glucose and glutamine metabolism that likely contributes to the antiproliferative effects of this immunosuppressive drug. The mechanism by which MPA promotes the metabolic reprogramming as well as the kinetics of the molecular events leading to the metabolic shutdown requires further clarification. However, the MPA-induced metabolic effects were not specific to the reduction of cell proliferation because Rapa, which also inhibits cell proliferation, produced distinct metabolic changes that were likely related to the immediate consequences of mTOR inhibition. Nevertheless, it is probable that multiple mechanisms are involved and that they interfere with one another. The decrease in intracellular ATP levels occurring very early after MPA exposure is probably related to the inhibition of the *de novo* synthesis of purines as well as a defect in the production of adenosine, since lymphocytes do not rely on the purine salvage pathway. As a consequence, the energy sensor AMPK is activated, and this may allow for the activation of compensatory mechanisms leading to ATP production through catabolic pathways, such as fatty acid beta-oxidation^40^. This transient effect of MPA on ATP production is different from the effect of Rapa, which produces a progressive reduction of ATP content over time. mTORC1 likely plays a critical role in these opposing effects because its inhibition by Rapa promotes mitochondrial dysfunction and a consequent reduction in OXPHOS (a highly efficient process for ATP production)^27^. MPA activates AMPK but does not inhibit mTORC1, which may explain why ATP can still be produced. Consistent with the efficiency of compensatory mechanisms during MPA exposure leading to the transient energetic stress, autophagy is not induced by MPA, although it is induced by Rapa exposure.

Conversely, MPA and Rapa share a common effect in reducing glycolytic and glutaminolytic fluxes. The shutdown of glycolysis reduces the production of pyruvate, and a reduction in glutaminolysis leads to the lack of α-ketoglutarate. Both of these result in the reduction of the activity of the TCA cycle and therefore reduce ATP production. In addition, the loss of glycolytic activity decreases the availability of metabolic intermediates that under normal circumstances, fuel anabolic pathways such as the pentose phosphate pathway (glucose-6-phosphate) and the serine biosynthesis pathway (3-phosphoglycerate), thereby reducing the ability of the cell to sustain growth and proliferation. The inhibition of glucose uptake in response to MPA and Rapa likely participates in the inhibition of glycolysis but does not involve a reduction in the expression of glucose transporters, suggesting that the activity of theses transporters could be affected instead. In line with this, our findings highlight the limitations of transcriptome analysis for the interpretation of the potential functional consequences of the administration of the drugs. Indeed, we observed a reduction in the expression of Myc and HIF-1α, which regulate many genes involved in glycolysis and glutaminolysis^41, 42^, such glucose and amino acid transporters, as well as genes involved in the production of pyruvate. On the other hand, our data showed that the expression of transcripts of target genes such as PFKBF3 (6-phosphofructo-2-kinase/fructose-2:6-biphosphatase-3) or Hexokinase II were upregulated and that glycolytic flux was decreased. These contradictory observations can be explained in two ways: (1) HIF-1α and Myc regulate the expression and activity of genes involved in glycolysis and glutaminolysis at the post-transcriptional level^43–45^, but the presence of mRNA transcripts does not imply that the gene product is functional; and (2) redundant pathways, such as transcription factors under the control of AKT, which remains activated by MPA, can regulate the expression of these genes despite the reduction in the expression of HIF-1α and Myc.

It is worth noting that the effects of MPA on metabolic checkpoints may vary according to the cellular model. A genome-wide transcriptome analysis showed that MPA modifies the activities of Myc and HIF-1α signalling pathways in endothelial cells, which can explain the antiangiogenic and potential antitumor effects of MPA^1, 23^. In a transcriptome profiling analysis, MPA affected the proliferation of cancer gastric cells in a PI3K-AKT-mTOR pathway-dependent manner^1, 24^, and in a gastric adenocarcinoma cell line, MPA decreased the activity of AKT and mTOR after 48 h and 72 h of treatment. However, in a model of androgen-sensitive human prostate cancer, MPA decreased the expression of Myc at 6 h and 24 h^46^, supporting our results. The Jurkat T cell line is a validated model for studying immunometabolism^27–30^ and for the biochemical characterization of T cell activation and signal transduction^47, 48^. Experiments performed to compare the metabolic characteristics of Jurkat T cells with that of activated peripheral blood T cells indicated that differences between these two cell models are minor^48–51^.

In conclusion, this study provides insights into the metabolic mechanisms driving the antiproliferative activity of MPA on Jurkat T cells. Considering the role of the immunometabolism in the polarization of T cells, our findings raise interesting issues regarding the impact of MPA on T cell activation phenotypes. Supporting this, Rapa, by inhibiting mTOR, decreases glycolysis and promotes the differentiation of memory CD8^+^ T cells and the generation of regulatory T cells both *in vitro* and *in vivo* ^52, 53^. Since the combined inhibition of glycolysis and mTORC1 signalling can disrupt metabolic reprogramming in tumour cells to inhibit their growth, these findings reveal potential benefits of novel combinatorial therapeutic strategies by co-targeting metabolic checkpoints to block lymphocyte proliferation or modulate cell differentiation during immunosuppressive treatments.

## Methods

### Cell culture and Chemicals

Jurkat T leukemia cells (clone E6–1, Lot Number 60628582, received November 2014) were purchased from the American Type Culture Collection (ATCC, Manassas, VA, USA). The Jurkat cells were cultured at 37 °C in RPMI 1640 medium (ref. A10491-01 from Gibco®, Thermo Fisher Scientific, Waltham, MA, USA) supplemented with 10% foetal bovine serum (FBS), 50 U/mL of penicillin and 50 μg/mL of streptomycin. The cell cultures were maintained at a density of 5 × 10^5^ cells/mL. This cell line was mycoplasma-free as tested with the Mycoalert Mycoplasma Detection Kit (Lonza, Slough, UK). MPA was purchased from Sigma-Aldrich (Rocky Hill, NJ, USA). Rapamycin was purchased from LC Laboratories (Woburn, MA, USA).

### Viability studies

Jurkat cells were seeded in 96-well plates (5 × 10^5^ cells/mL), and the relative number of live cells per well was determined by using the Cell Titer 96® Aqueous One Solution Cell Proliferation Assay with 3-(4,5-dimethylthiazol-2-yl)-5-(3-carboxymethoxyphenyl)-2-(4-sul-fophenyl)-2H-tetrazolium (MTS) (Promega, Madison, WI, USA) according to the manufacturer’s protocol.

### Cell apoptosis assay

The cell apoptosis assay was performed as described previously^54^. The Jurkat cells were seeded in 12-well plates (5 × 10^5^ cells/mL) and incubated with 0.5 μM MPA, 5 μM rapamycin or vehicle (ethanol, V) for 24 or 48 h. After treatment, apoptosis was analysed by combining 25 µL of cell suspension with 25 µL of a mixture containing ethidium bromide (EB, 500 μg/mL) and acridine orange (AO, 150 μg/mL). Cell morphology was studied using a fluorescence microscope. Approximately 100 to 200 cells were counted per condition. The live cells were stained green, and the apoptotic cells were stained orange with shrunken and fragmented nuclei. Among all the cells counted, the percentage of apoptotic cells was calculated.

### RNA extraction and quantitative real-time polymerase chain reaction (qRT-PCR)

RNA extraction and qRT-PCR was performed as previously reported^55^. In short, total RNA was extracted using the RNeasy Mini Kit® (Qiagen, Valencia, CA, USA) according to the manufacturer’s protocol. The mRNA expression levels were assessed by using a SYBR Green qRT-PCR kit with an ABI-PRISM 7900 sequence detector system (Applied Biosystems, Foster City, CA, USA). The fold-changes for each tested gene were normalized to the housekeeping gene ribosomal protein L13A (RPL13A). The relative expression of each gene was calculated using the 2^−ΔΔCT^ method^56^. Consequently, the expression level of a given gene in the control samples (vehicle-treated) using the 2^−ΔΔCT^ method was set to 1. The results were visualized as heat maps. The primer sequences are listed in Supplementary Table 1.

### Protein extraction and Western blot analysis

Immunoblotting was performed as previously described^55^. Total protein lysates were separated by sodium dodecyl sulfate polyacrylamide (SDS-PAGE) gel electrophoresis under denaturing conditions and transferred to polyvinylidene fluoride (PVDF) membranes (GE Healthcare, Pittsburgh, PA, USA). The primary antibodies targeting relevant proteins (listed in Supplemental Table 2) were visualized using horseradish peroxidase-conjugated polyclonal secondary antibodies (Cell Signaling Technology Inc. (Hitchin, UK) and detected with an ECL reagent® (GE Healthcare).

### ATP detection assay

Jurkat cells (10^3^ cells per well) were plated in 96-well plates, and the ATP levels were analysed using an ATPlite 2 steps Kit (PerkinElmer, Waltham, MA, USA) according to the manufacturer’s protocol. Luminescence was measured with a microplate luminescence counter Enspire Multilabel Reader 2300 (Perkin Elmer).

### Detection and quantification of purine and pyrimidine metabolites

Metabolite extraction was achieved by resuspension of pellet cells (5 millions cells per condition) with 100 µl H20 at 0 °C. Next, samples were maintained for 10 min at 100 °C and centrifuged for 10 min at ~13 000 g. The supernatants were collected for 1- measurements of purine and pyrimidine metabolites by an Agilent 1290 infinity HPLC system coupled with Diode array detector as recommended by the ERDNIM advisory document^57^ and 2- total protein content with the kit “Pierce^TM^ BCA Protein Assay Kit” (Thermo Scientific). Metabolites were separated using C18 Nucleosil column (250mm length, 4.6mm diameter; Interchim). Each Metabolite amount was normalized to protein content.

### Lactate measurements

Extracellular lactate levels were analysed by using the Lactate Colorimetric/Fluorometric Assay Kit (BioVision Inc., Milpitas, CA, USA) in Krebs phosphate buffer (at pH 7.6, 6.3 g/L NaCl, 320 mg/L KCl, 140 mg/L CaCl_2_, 148 mg/L KH_2_PO_4_, 267 mg/L MgSO_4_ and 1.91 g/L NaHCO_3_) according to the manufacturer’s protocol.

### Metabolic assays

Glucose and glutamine oxidation fluxes were determined by the rate of ^14^CO_2_ released from ^14^C-U-glucose and ^14^C-U-glutamine, respectively. The Jurkat cells were treated for 48 h with 0.5 μM MPA or 5 μM rapamycin. Then, 5 × 10^6^ cells were resuspended in 950 μL of Krebs-Ringer phosphate buffer supplemented with either 5 mM ^14^C-U-glucose (11 GBq/mmol, isotopic dilution 1/1000, Perkin Elmer) or 4 mM ^14^C-U-glutamine (9.69 GBq/mmol, isotopic dilution 1/1000, Perkin Elmer). After a 90-min incubation at 37 °C, the reaction was stopped by adding 250 μL of 6 N H_2_SO_4_, and CO_2_ was recovered for 1 h in benzethonium hydroxide. The radioactive CO_2_ was quantified by using liquid scintillation (Ultima Gold, Perkin Elmer).

### Glucose uptake

Glucose uptake was analysed according to the protocol described by Hardonnière *et al*.^58^ with some adjustments. After the treatment of the cells with 0.5 μM MPA or 5 μM rapamycin for 48 h, 5 × 10^6^ cells were washed with PBS and incubated in 5 mL of glucose-free RPMI 1640 medium supplemented with 10% SVF and 2 mM of glutamine at 37 °C for 3 h. After this starvation period, the cells were washed and incubated with Krebs-Ringer phosphate buffer for 30 min at 37 °C followed by an incubation with 0.1 mM ^3^H-2-deoxyglucose (isotopic dilution of 1:4000) for 10 min at 37 °C. After the cells were gently washed twice with ice-cold Krebs-Ringer phosphate buffer, the cell pellets were lysed by adding 250 μL of 0.1 N NaOH. Half of the sample content was transferred into scintillation vials, and the radio-labelled glucose incorporated into the cells was measured by using Ultima Gold and reading the samples on a liquid scintillation counter. The protein content for each condition was assayed by using the remaining half of the sample with a Pierce^TM^ BCA Protein Assay Kit (Thermo Fisher Scientific).

### Statistical Analysis

The results are expressed as the means ± SD. The distributions are represented using histograms, and the distribution of variables are represented with dots plots. We used the Mann-Whitney U test for nonparametric data comparisons between two groups and the t-test to compare the parametric data. Statistical analyses were performed using GraphPad Prism software version 5.0 (GraphPad Software Inc., La Jolla, CA, USA), which was also used to generate the graphs. P-values < 0.05 were considered statistically significant.

### Data availability

All data generated during and/or analysed during the current study are available from the corresponding author on reasonable request.

## Electronic supplementary material

Supplementary information
