# Supplementary material for: A comprehensive characterization of the impact of mycophenolic acid on the metabolism of Jurkat T cells
Source: Sci Rep. 2017 Sep 5;7:10550. doi: 10.1038/s41598-017-10338-6 (PMC5585210; doi:10.1038/s41598-017-10338-6)
Supplement: Supplementary file 1 — Supplementary information [file 41598_2017_10338_MOESM1_ESM.pdf]

A comprehensive characterization of the impact of mycophenolic acid on the metabolism of proliferating T cells

Ana A. Fernández-Ramos,[1,2] Catherine Marchetti-Laurent,[1,2] Virginie Poindessous,[1,2] Samantha Antonio,[2,3] Céline Petitgas,[2,5] Irène Ceballos-Picot,[2,5] Pierre Laurent-Puig,[1,2,4] Sylvie Bortoli,[2,3] Marie-Anne Loriot,[1,2,4‡] Nicolas Pallet[1,2,4‡*]

Supplementary Figure 1

Figure S1. Full-length blots of p70s6k, Myc, HiF-1α and actin after 24 and 48 hours of incubation. A) Western Blot of p-p70s6k and actin after incubation for 24 hours with V (1st column), MPA (3rd column) and Rapa (4th column). B) Western Blot of HIF-1α, Myc and actin after incubation for 24 hours with V (1st column), MPA (3rd column) and Rapa (4th column). C) Western Blot of p-p70s6k and actin after incubation for 48 hours with V (1st column), MPA (3rd column) and Rapa (4th column). D) Western Blot of HIF-1α, Myc and actin after incubation for 48 hours with V (1st column), MPA (3rd column) and Rapa (4th column).

A)

24h

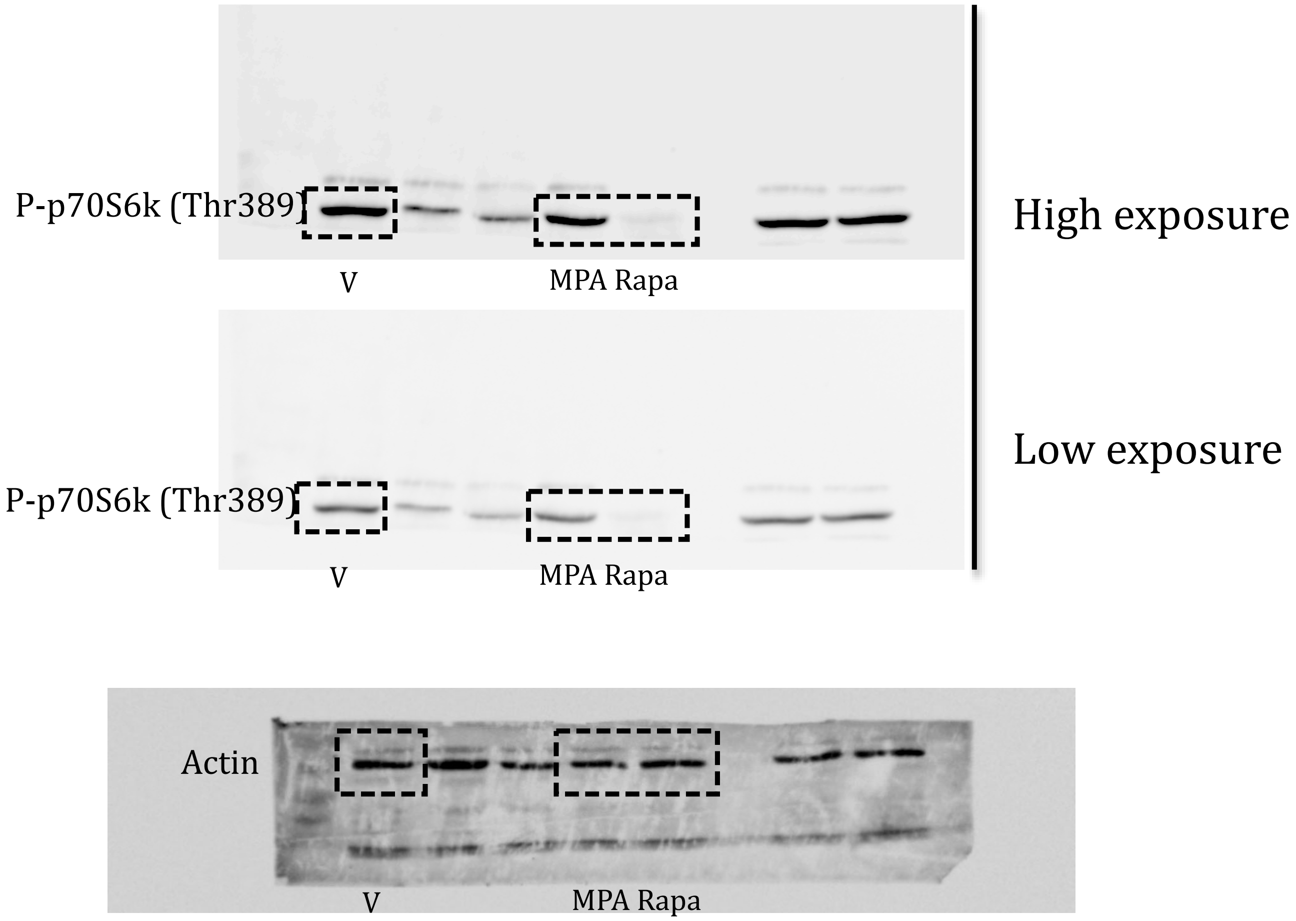

B)

24h

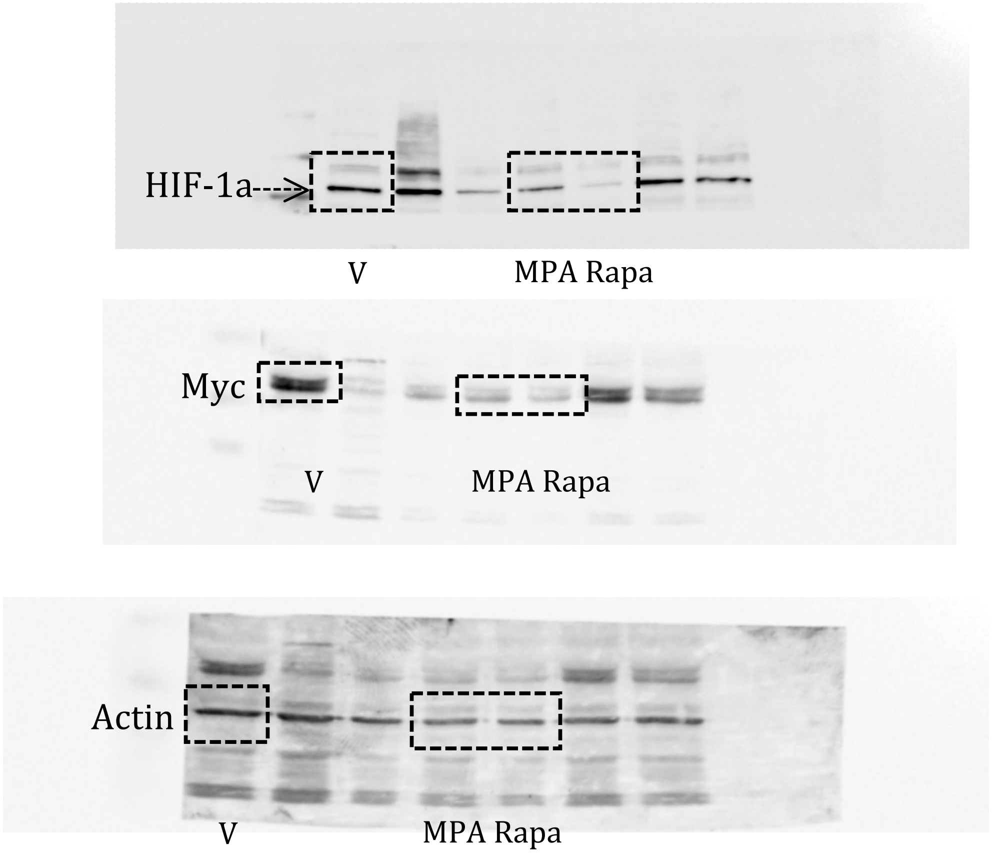
HIF-1a
V
MPA Rapa
Myc
V
MPA Rapa
Actin
V
MPA Rapa

C)

48h

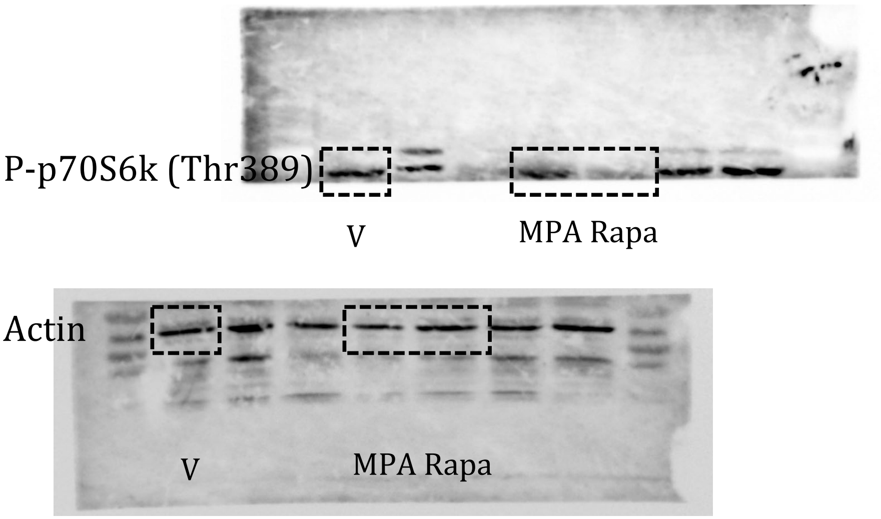
P-p70S6k (Thr389)
V
MPA Rapa
Actin
V
MPA Rapa

D)

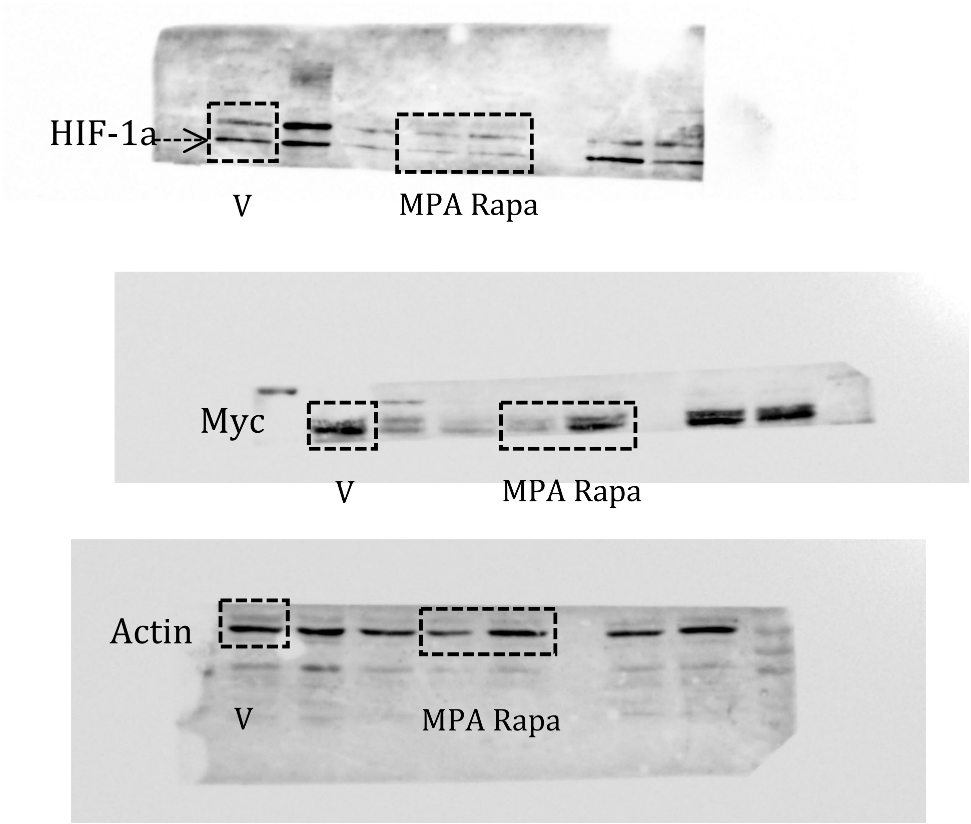

Supplementary Figure 2

Figure S2. MPA inhibits guanine, GMP and GTP or GDP but not pyrimidines. High Performance Liquid Chromatography was used to profile purines and pyrimidines molecules in T cells. Values were normalized by mg of proteins of intracellular levels of guanine, GTP/GDP (no differentiation by HPLC), CTP/CDP (no differentiation by HPLC), UTP, UDP, fumarate, SAM (S-adenosylmethionine) and nicotinamide after 2 h, 4 h, 6 h and 16 hours of incubation with vehicle (V, black) or 0.5 µM MPA (white). The data is from three independent experiments.

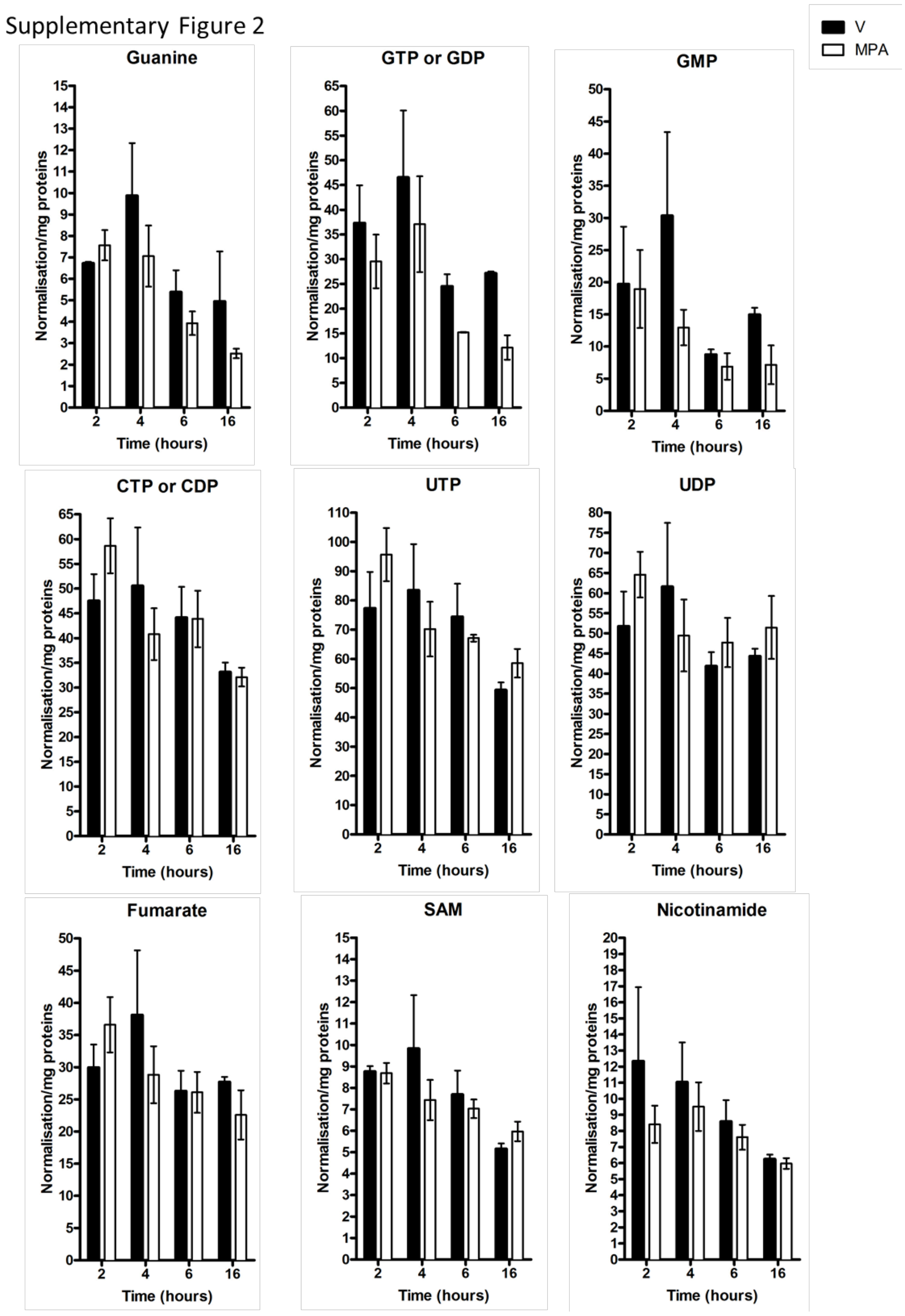

Supplementary Methods

Supplementary Table 1. Primer sequences used for RT-PCR analysis

Gene name	Primer sequence
GPI	F 5'-ccaccagcagacacacatca-3'<br>R 5'-cctgtgcactagtcggcttc-3'
HK2	F 5'-tggcagaccttcatcttccttc-3'<br>R 5'-aaacacacagtggaaactggc-3'
PFKFB3	F 5'-ggtgtgcgacgaccctac-3'<br>R 5'-gtacacgatgcggctctg-3'
PKM	F 5'-cgggataaccttgaggctga-3'<br>R 5'-gaagagatccggagccacg-3'
SLC2A1	F 5'-tatgtggagcaactgtgtggt-3'<br>R 5'-tccggcctttagtctcagga-3'
SLC2A3	F 5'-gacacagaaggtcaccccag-3'<br>R 5'-gacgaagagtccgacggaaa-3'
ENO1	F 5'-ttcggctcaccggtcctatc-3'<br>R 5'-ggagagccgtcactcattcc-3'
TPI	F 5'-cgttgggggaaactggaagat-3'<br>R 5'-aaaccacctcggtgtcggc-3'
LDHA	F 5'-acgtcagcatagctgtgcaa-3'<br>R 5'-aggaatcgggaatgcacgtc-3'
LDHB	F 5'-gcctcctctctcctgtgcaa-3'<br>R 5'-cctcttcttccgcaactggt-3'
MCT1	F 5'-ggttataaggcagcctcgc-3'<br>R 5'-ttgcttgttccagtacccacg-3'
GLS1	F 5'-tccccaaggacaggtggaat-3'<br>R 5'-gaggtgtgtggacttggt-3'
SLC1A5	F 5'-tggactggctagtcgaccg-3'<br>R 5'-gggcagctcactcttcactt-3'
SLC38A1	F 5'- gggattttgggactcgcctt-3'<br>R 5'- tacaccatgcagcctgtttct-3'
SLC3A2	F 5'- gggcctggactcttctccta-3'<br>R 5'- ggcccacatccccaaagtta-3'
GLUD1	F 5'- gtaactgcatggcttaacctgg-3'<br>R 5'- tctgggcagctcacaataaag-3'
HGPRT	F 5'- ttgctttccttggtcaggca-3'<br>R 5'- atccaacacttcgtggggtc-3'
IMPDH1	F 5'- gtctgtccttcggtccatga-3'<br>R 5'- gccgcttttcgtaagagtgc-3'
IMPDH2	F 5'-tggaggcaatgtggtcactg-3'<br>R 5'-gccagcacttcctgcgtaa-3'
CAD	F 5'- ggagtttgcagtccttcccg-3'<br>R 5'- gccggtttgaaacaccactt-3'
G6PD	F 5'-cgacgacgacgacgaagcgcaga-3'<br>R 5'-ggccaggtcacccgatgcac-3'
TKT	F 5'-tgtgtccagtgcagtagtgg-3'<br>R 5'-acacttcatacccgccctag-3'
RPL13A	F 5'-cctggaggagaagaggaaagaga-3'<br>R 5'-gaggacctctgtgtatttgtcaa-3'

Supplementary table 2. Primary antibodies used for Western blot

**Antibody**	**Dilution**	**Reference**
Anti-β actin	1:10000	A2668[1]
Anti-Phospho-p70 S6 Kinase (Thr421/Ser424)	1:1000	9204[2]
Anti-HIF-1 alpha	1:1000	NB100-449[3]
Anti-c-Myc	1:1000	5605[2]
Anti-AMPKα	1:1000	2532[2]
Anti-Phospho-AMPKα (Thr172)	1:1000	2535[2]
Anti-Glut1	1:1000	NB110-39113[3]
Anti-Glut3	1:1000	NBP1-89762[3]
Anti-LC3B	1:2000	2775[2]

[1] Sigma-Aldrich
[2] Cell Signalling Technology
[3] Novus Biologicals
